# Secukinumab in active lupus nephritis: results from a phase III randomized, placebo-controlled study (SELUNE) and an open-label extension study

**DOI:** 10.1093/rheumatology/keaf536

**Published:** 2025-10-15

**Authors:** Ming-hui Zhao, Fidencio Cons Molina, Gustavo Aroca, Maria G Tektonidou, Anubhav Mathur, Radhika Tangadpalli, Rui Sun, Ruvie Martin, Pascale Pellet, Thao Ngoc Phuong Huynh

**Affiliations:** Renal Division, Peking University First Hospital, Peking University Institute of Nephrology, Beijing, China; Centro de Investigación en Artritis y Osteoporosis, Mexicali, Mexico; Renal Division, Simon Bolivar University, Barranquilla, Colombia; Rheumatology Unit, Joint Academic Rheumatology Program, First Department of Propaedeutic and Internal Medicine, National and Kapodistrian University of Athens, Athens, Greece; Novartis Pharmaceuticals Corporation, East Hanover, NJ, USA; Novartis Healthcare Private Limited, Hyderabad, India; Novartis Pharmaceuticals Corporation, Bannockburn, IL, USA; Novartis Pharmaceuticals Corporation, East Hanover, NJ, USA; Novartis Pharma AG, Basel, Switzerland; Nephrology and Hemodialysis Department, University Medical Center Ho Chi Minh City, University of Medicine and Pharmacy at Ho Chi Minh City, Ho Chi Minh City, Vietnam

**Keywords:** biologic therapies, clinical trials and methods, interventional studies, renal, systemic lupus erythematosus, autoimmunity

## Abstract

**Objective:**

The objective of this study was to evaluate the efficacy and safety of s.c. secukinumab 300 mg combined with standard of care (SoC) in adult patients with active LN.

**Methods:**

The core study was a phase III randomized, placebo-controlled, double-blind study (planned duration: 104 weeks) of patients with active LN. Eligible patients were randomized to receive secukinumab 300 mg or placebo for the first 4 weeks, followed by a monthly maintenance dose, with all patients receiving a SoC background regimen. Patients completing the 104-week treatment of the core study were eligible for an open-label extension study (planned duration: up to 260 weeks) to receive secukinumab 300 mg every 4 weeks. The primary end point of the core study was the proportion of patients achieving complete renal response (CRR) at week 52.

**Results:**

Both studies were terminated early, following a planned futility analysis in the core study that showed no clinically meaningful benefit of secukinumab over placebo. In the core study, the proportion of patients achieving CRR at week 52 was lower after receiving secukinumab (24.2%) than after receiving the placebo (36.3%). No differences were observed between the secukinumab and placebo groups in any of the secondary end points of the core study. The incidence of treatment-emergent adverse events was comparable between the secukinumab and placebo groups. In the extension study, the results were not interpretable, owing to the low patient number included in the data analysis (*n* = 31).

**Conclusion:**

Secukinumab did not demonstrate superior efficacy over placebo in patients with active LN. Secukinumab was well-tolerated with no new or unexpected safety signals detected.

**Trial registration:**

https://clinicaltrials.gov/study/NCT04181762, NCT04181762 (Novartis study code: CAIN457Q12301). https://www.clinicaltrials.gov/study/NCT05232864, NCT05232864 (Novartis study code: CAIN457Q12301E1).

Rheumatology key Messages

- Managing LN remains challenging, with a high risk of kidney failure, highlighting unmet treatment needs.
- Secukinumab did not improve complete renal response in patients with active LN compared with placebo.
- The safety profile of secukinumab in patients with LN was consistent with the approved indications.

## Introduction

LN occurs in ∼50% of patients with SLE and is associated with significant morbidity and mortality [1]. The current management of LN is based on disease severity and includes CSs, anti-malarial agents, and CS-sparing immunosuppressive agents. Despite the availability of new therapeutic options, the trials and observational studies have shown a complete renal response (CRR) of <50%, and the long-term effects of these therapeutic options are not yet known [2]. Therefore, LN management remains challenging. Without effective control of disease activity, patients may develop end-stage kidney disease (ESKD) and ultimately need renal replacement therapy [1, 3]. The evidence suggests that 6% to 19% of patients with LN develop ESKD over 10 years [3–5]. Recently, the Lupus Midwest Network (LUMEN) registry revealed that the survival rate for LN was 70%, and that 13% of patients with LN developed ESKD at 10 years [4]. Therefore, even with the current treatments, the risk of kidney failure remains high, highlighting the unmet treatment need in LN. In addition, factors such as limited access to specialized lupus centres and poor treatment adherence significantly impact outcomes in LN. These barriers contribute to delayed diagnosis and suboptimal disease control, underscoring the need for holistic management strategies [6].

The pathophysiology of LN is complex and is characterized by autoantibody production and inflammatory cell infiltration into renal tissues. IL-17–producing Th type 17 (Th17) cells exhibit significant hyperactivation, leading to inflammation, which has been implicated in LN-associated kidney damage [7, 8]. Moreover, IL-17 stimulates inflammatory cytokine production by renal cells, leading to granulopoiesis as well as changes in renal function [9]. Thus, IL-17 may contribute to disease progression, and its inhibition may lead to clinical improvement in LN [10].

Secukinumab, an anti–IL-17A antibody, has shown efficacy with a consistent and favourable safety profile in psoriasis, PsA, axial SpA, and hidradenitis suppurativa [11, 12]. Previous case reports have suggested improvement in LN with secukinumab [13, 14]. A patient with refractory LN achieved a CRR within 8 months of secukinumab treatment [13].

A phase III core study (SELUNE) and an extension study were conducted to evaluate the efficacy and safety of s.c. secukinumab 300 mg compared with placebo, in combination with the standard of care (SoC) for patients with active LN. Both studies were terminated early due to futile results following a planned futility analysis of the core study. The final results of the two studies are reported herein.

## Methods

### Study design and population

SELUNE was a multicentre phase III, randomized, placebo-controlled, double-blind study (planned duration: 104 weeks). The extension open-label study involved only secukinumab (planned duration: up to 260 weeks). The details of the study designs are presented in Supplementary Figure S1.

Eligible patients aged 18–75 years with a confirmed diagnosis of SLE and documented history of ≥4 of the 11 criteria for SLE as defined by the ACR (or LN as the sole clinical criterion in the presence of ANA or anti-dsDNA antibodies) and with active LN were included in the core study. Patients who completed the treatment period for up to week 104 of the core study were eligible to participate in the extension study. Detailed eligibility criteria are provided in Supplementary Table S1.

The core study planned to enrol 400 patients, who were randomized 1:1 to receive either s.c. secukinumab 300 mg or placebo for the first 4 weeks followed by a monthly maintenance dose thereafter. All patients received a SoC background regimen for induction [CSs along with either mycophenolic acid (MPA) or CYC], followed by maintenance therapy (MPA and oral CS). Patients entering the extension study received open-label secukinumab 300 mg every 4 weeks.

The SELUNE (core) study was registered (NCT04181762), as was the extension study (NCT05232864).

### End points

The primary end point was the proportion of patients achieving CRR with secukinumab compared with placebo at week 52 (core study) and at week 260 (extension study).

The secondary end points of the core study at week 52 included change from baseline in 24-hour urine protein-to-creatinine ratio (UPCR), proportion of patients achieving partial renal response (PRR), average daily dose of oral CSs between weeks 16 and 52, time to achieve CRR and PRR, time to achieve first morning void UPCR of ≤0.5 mg/mg, improvement in patient-reported outcomes [Functional Assessment of Chronic Illness Therapy–Fatigue (FACIT-F), Short Form Health Survey-Physical Component Summary (SF-26 PCS) and LupusQoL]. Safety was assessed by clinical laboratory evaluations, vital signs, and monitoring for treatment-emergent adverse events (TEAEs) in both studies.

### Statistical analysis

The core study had a pre-planned futility analysis when 138 patients had completed treatment up to week 52. An independent external data monitoring committee (DMC) reviewed the analysis. This futility analysis showed that no clinically meaningful benefit of secukinumab over the current SoC was expected to be observed by the end of the core study, based on the pre-specified futility criteria. Due to these findings of treatment futility, both the core and extension studies were terminated early. The final analysis was performed on patients who had or would have had an opportunity to reach 52 weeks of treatment at the time of core study termination (including those patients who discontinued the study treatment). A logistic regression model adjusted for SoC, race, and baseline UPCR was used for the primary analysis in the core study. The difference in marginal response proportions, along with the *P*-value and 95% CI), was estimated. The significance level was set at 5% (two-sided). The secondary efficacy analyses have been summarized with descriptive statistics using the observed data. Due to early termination, none of the patients completed the extension study, and only the available efficacy results are presented with the summary statistics. Further details of the statistical procedures are presented in the Supplementary appendix.

## Results

### Patient disposition and demographics

Overall, 275 patients were randomized to receive either secukinumab (*n* = 137) or a placebo (*n* = 138) in the core study. Of these patients, 182 (91 in each group) were included in the final analysis (i.e. those patients who had a chance to reach week 52 at the time of the study termination). Among the latter group, 71.4% of the secukinumab group (65/91) and 74.7% of the placebo group (68/91) completed the double-blinded 52-week treatment period. At week 104, 31 patients moved from the core to the extension study (Supplementary Table S2). The baseline demographics and disease characteristics were generally balanced between the groups. At the core study baseline, the mean patient age was 33.6 years, and most patients were female (87.3%). Overall, 48.4% of patients had LN class IV without class V features. The mean (s.d.) time since first diagnosis of LN was 3.1 (4.7) years (Supplementary Table S3).

### Efficacy

#### Core study

##### Primary end point

Based on the available data, the core study did not achieve the primary objective. At week 52, the proportion of patients achieving CRR was lower with secukinumab (24.2%) than with placebo (36.3%). The estimated mean response rates at week 52 were numerically lower with secukinumab (25.9%; 95% CI: 16.8–34.9) than with placebo (38.6%; 95% CI: 28.5–48.7). The number of responders for all individual CRR components was lower in the secukinumab group than in the placebo group, except for the estimated glomerular filtration rate (for which it was comparable) (Table 1). The main contributor to the numerically lower number of responders in the secukinumab group compared with the placebo group was the 24-hour UPCR of ≤0.5 mg/mg.

**Table 1.** Proportion of patients achieving CRR in core study

Core study						
Analysis visit	Response (using NRI)^b^	SEC (*N* = 91)	Placebo (*N* = 91)			Estimated mean response rate difference (CI)
Week 52—Primary^a^	Responder, *n* (%)	22 (24.2)	33 (36.3)			
	Estimated mean response rate (95% CI)	25.9 (16.8, 34.9)	38.6 (28.5, 48.7)			−12.7 (−26.3, 0.9)
	**CRR components, *n* (%)**					
	24-h UPCR ≤ 0.5 mg/mg	27 (29.7)		40 (44.0)		
	eGFR ≥ 60 ml/min/1.73 m^2^ or not less than 85% of baseline	68 (74.7)		67 (73.6)		
	No treatment discontinuation^c^ before week 52	66 (72.5)		69 (75.8)		
	No CS overuse between week 44 and week 52	84 (92.3)		90 (98.9)		

a The week 52 primary analysis considered patients with CS overuse between week 44 and week 52 as non-responders. CS overuse was defined as receipt of >10 mg prednisone or equivalent for ≥3 consecutive days or for ≥7 days of total during weeks 44 through 52.

b Patients who did not have the required data for computing responses at the specific time point or who had discontinued the study treatment before the specified time point were classified as non-responders.

c Treatment discontinuation for reasons other than early study termination by sponsor.

CRR: complete renal response; eGFR: estimated glomerular filtration rate; *N*: total number of patients; *n*: number of assessable patients; NRI: non-responder imputation; SEC: secukinumab; UPCR: urine protein-to-creatinine ratio.

##### Secondary end points

The PRR at week 52 was lower in the secukinumab group (56.2%) than in the placebo group (63.9%). Secukinumab *vs* placebo groups showed similar changes from baseline at week 52 in mean FACIT-F scores (−2.0 *vs* −2.0), SF-36 PCS scores (3.410 *vs* 2.707) and LupusQoL scores (7.62 *vs* 8.55). The other secondary end point results are presented in Supplementary Table S4.

#### Extension study

In the extension study, due to fewer patients being included in the data analysis (*n* = 31), the results were not interpretable (Supplementary Table S5).

### Safety

The overall incidence of TEAEs was comparable between the treatment groups in the core (secukinumab: 87.6%; placebo: 89.1%) and extension (secukinumab: 93.8%; placebo–secukinumab: 100.0%) studies.

During the core study, the most frequently reported TEAEs (≥10%) were comparable between the groups, including upper respiratory tract infection, COVID-19, urinary tract infection (UTI), arthralgia, diarrhoea, headache, and cough (Table 2).

**Table 2.** Summary of treatment-emergent adverse events in core study and extension study

Core study		
	SEC (*N* = 137)	Placebo (*N* = 138)
Exposure (days), mean (s.d.)	455.7 (229.6)	443.4 (232.9)
Exposure (days), median (min–max)	421.0 (1–786)	423.5 (44–794)
Patient years	171	168
Any AEs, *n* (%)	120 (87.6)	123 (89.1)
Death, *n* (%)	1 (0.7)	1 (0.7)
Any SAEs, *n* (%)	30 (21.9)	39 (28.3)
Discontinued study treatment due to any AEs, *n* (%)	8 (5.8)	10 (7.2)
Any AEs by severity, *n* (%)		
Mild	46 (33.6)	50 (36.2)
Moderate	60 (43.8)	54 (39.1)
Severe	14 (10.2)	19 (13.8)
Most common AEs by preferred term (≥10%), *n* (%)		
Arthralgia	13 (9.5)	19 (13.8)
Cough	8 (5.8)	14 (10.1)
COVID-19	28 (20.4)	25 (18.1)
Diarrhoea	23 (16.8)	21 (15.2)
Headache	21 (15.3)	14 (10.1)
URTI	19 (13.9)	26 (18.8)
UTI	19 (13.9)	31 (22.5)
AEs of special interest by preferred term^a^ (≥5%), *n* (%)		
Gastroenteritis	7 (5.1)	8 (5.8)
Herpes zoster	10 (7.3)	8 (5.8)
Nasopharyngitis	11 (8.0)	11 (8.0)
Oral candidiasis	8 (5.8)	3 (2.2)
Pharyngitis	4 (2.9)	11 (8.0)
Pneumonia	9 (6.6)	3 (2.2)
Extension study^b^		
	SEC (*N* = 16) *n* (%)	Placebo-SEC (*N* = 15) *n* (%)
Exposure (days), mean (s.d.)	148.2 (72.2)	158.5 (81.0)
Exposure (days), median (min–max)	113.5 (85–281)	113.0 (83–310)
Patient years	6.5	6.5
Any AEs, *n* (%)	15 (93.8)	15 (100.0)
Death, *n* (%)	0	0
Any SAEs, *n* (%)	0	3 (20.0)
Any AEs by severity, *n* (%)		
Mild	3 (18.8)	7 (46.7)
Moderate	11 (68.8)	5 (33.3)
Severe	1 (6.3)	3 (20.0)
Most common AEs by preferred term (≥15%), *n* (%)		
Anaemia	4 (25.0)	3 (20.0)
Arthralgia	3 (18.8)	3 (20.0)
COVID-19	5 (31.3)	8 (53.3)
Diarrhoea	2 (12.5)	4 (26.7)
Headache	3 (18.8)	0
Herpes zoster	5 (31.3)	0
Hypokalaemia	1 (6.3)	3 (20.0)
Nausea	3 (18.8)	0
Pharyngitis	0	3 (20.0)
URTI	0	4 (26.7)
UTI	4 (25.0)	6 (40.0)
AEs of special interest by preferred term^a^ (≥10%), *n* (%)		
Bacterial vaginosis	2 (12.5)	1 (6.7)
Breast cancer	0	2 (13.3)
Nasopharyngitis	1 (6.3)	2 (13.3)
Paronychia	1 (6.3)	2 (13.3)

a AEs that are already covered under most common AEs were not included.

b The baseline of the safety data for the extension study was defined as the last measurement made prior to administration of the first dose of the study treatment in the core study.

COVID-19: coronavirus disease; AE: adverse event; *N*: total number of patients; *n*: number of assessable patients; SAE: serious adverse event; SEC: secukinumab; UTI: urinary tract infection; URTI: upper respiratory tract infection.

No major adverse cardiovascular events (MACEs) were reported in any patient receiving secukinumab in either the core or the extension studies.

In the extension study, the most frequently reported TEAEs (≥15%) were COVID-19, UTI and anaemia (Table 2). Most TEAEs reported were mild to moderate during both studies.

The incidence of serious adverse events (SAEs) was lower with secukinumab (21.9%) than with placebo (28.3%), with two deaths being reported during the core study (one patient in each group). One ((35-year-old) patient from the secukinumab group died due to meningitis on day 33. The investigator assessed the event as related to the study treatment and background SoC of MMF and CSs. This death was assessed by the study sponsor (Novartis Pharmaceuticals) as not being related to the study treatment but due to the background immunosuppressive treatment. The other (21-year-old) patient in the placebo group died of pericardial effusion and active SLE on day 205. During the core study, the SAEs suspected to be related to the study treatment were comparable across both treatment groups (Supplementary appendix). No deaths were reported during the extension study, and three SAEs were reported in the placebo–secukinumab group: two cases of breast cancer and one case of UTI.

The most frequent AEs of special interest were infections in both the core and extension studies (Table 2). The overall incidence of infections was similar between the groups. An imbalance in fungal infections was observed between secukinumab (12.4%) and placebo (5.8%) groups in the core study. These infections were mainly *Candida* infections (secukinumab: 7.3%; placebo: 2.9%), and oral candidiasis was the most common *Candida* infection (secukinumab: 5.8%, placebo: 2.2%). Additionally, oesophageal candidiasis was <1.5% in both treatment groups in the core study. Treatment discontinuations due to infections were numerically lower with secukinumab (0.7%) than with the placebo (1.4%) in the core study.

## Discussion

Despite recent advancements in the understanding of LN pathophysiology and the continuous development of novel agents, ∼60% of patients may not achieve a complete response, leading to poor long-term outcomes [15]. Moreover, a notable proportion of patients experience relapse and flares [16]. Therefore, there remains an unmet treatment need for achieving complete remission and preventing relapse and progression of LN to ESKD.

The core (SELUNE) and the extension studies aimed to evaluate the long-term efficacy and safety of secukinumab. However, both studies were terminated early at the recommendation of an independent DMC due to findings of a planned futility analysis of the core study showing no clinically meaningful benefit of secukinumab over the current SoC. There were no specific safety concerns that led to early termination of these studies. The results of the final analysis were aligned with the outcome of the futility analysis. The core study showed that the response rate of achieving CRR was numerically lower with secukinumab than with placebo. No differences were observed between the secukinumab and placebo groups in any of the secondary efficacy end points, including patient-reported outcomes. Due to early termination of the studies, the long-term efficacy of secukinumab in LN could not be evaluated.

The incidence of TEAEs was comparable between the secukinumab and placebo groups, except for herpes zoster and fungal infections (mainly oral candidiasis) with secukinumab, which were non-serious and mild to moderate in severity and in line with the known safety profile of secukinumab.

No MACE was reported in the secukinumab-treated patients in this population, even though this population is known to have an increased risk of LN-associated cardiovascular events [17]. Safety results were consistent with the known safety profile of secukinumab in the approved indications with no new or unexpected safety signals [12].

Several biologic molecules, including ianalumab, daratumumab, obinutuzumab (shown to be effective over placebo in achieving CRR), guselkumab, and anifrolumab, are being investigated for their efficacy and safety in patients with LN [18, 19]. Despite the ongoing efforts and numerous randomized controlled trials (RCTs), a significant number of RCTs have also failed. The factors to which these failures have been attributed include trial design, sample size, eligibility criteria, end point definition, outcome measures, disease heterogeneity, strong medication background, or short follow-up [20]. The core (SELUNE) study had generally balanced baseline characteristics and consistent background SoC in both treatment arms, with patients stratified at randomization by induction SoC therapy to ensure balanced representation and to minimize confounding from patient selection or induction of treatment variability. Despite this, secukinumab did not show superiority over placebo. In another RCT, LUNAR, rituximab did not show added benefit over SoC in LN, despite effective B cell depletion [21]. In both LUNAR and SELUNE studies, high placebo response rates and a stringent end point definition, particularly the proteinuria threshold for CRR, may have reduced the sensitivity for detecting a marginal treatment response. Strong background SoC therapy may also have contributed to the high placebo response. However, the lack of efficacy with secukinumab, despite a robust RCT, suggests that IL-17A inhibition may not benefit the broader LN population. These challenges highlight the complexities of drug development in LN, where addressing only the inflammatory process of its multifactorial pathophysiology may not be adequately effective.

The secukinumab dosing regimen was selected based on its established efficacy and safety in other inflammatory diseases. However, population PK modelling showed lower drug exposure between weeks 12 and 24 in patients with LN than in those with psoriasis and PsA, likely due to proteinuria-driven drug clearance (Novartis data on file). Nonetheless, exposure levels in patients with LN remained higher than those achieved with 150 mg secukinumab administered every 4 weeks, which is effective in other indications. Therefore, while PK differences may have contributed marginally, they are unlikely to fully explain the lack of efficacy. These findings further support the hypothesis that IL-17A–targeted therapy may not be appropriate for LN.

Due to the early termination of both studies, the small sample size and an incomplete follow-up, only limited inferences could be drawn from the results.

## Conclusion

Secukinumab combined with SoC did not demonstrate superior efficacy to that of placebo with SoC in patients with active LN. The safety profile of secukinumab was consistent with the known safety profile in approved adult indications and showed no new or unexpected safety signals in patients with LN.

The study protocol and all amendments were reviewed and approved by the Independent Ethics Committee/Institutional Review Board of each centre. The study was conducted according to the International Council for Harmonisation E6 Guidelines for Good Clinical Practice, which have their origin in the Declaration of Helsinki. Informed consent was obtained from each patient in writing at the screening visit.

## Supplementary Material

keaf536_Supplementary_Data

## Data Availability

The datasets generated and/or analysed during the current study are not publicly available but may be made available upon reasonable request. Novartis is committed to sharing access to patient-level data and supporting clinical documents from eligible studies with qualified external researchers. These requests are reviewed and approved on the basis of scientific merit. All data provided are anonymized to respect the privacy of patients who have participated in the trial in line with applicable laws and regulations. The data may be requested from the corresponding author of the manuscript.
