# Supplementary material for: Secukinumab in active lupus nephritis: results from a phase III randomized, placebo-controlled study (SELUNE) and an open-label extension study
Source: Rheumatology (Oxford). 2025 Oct 15;65(1):keaf536. doi: 10.1093/rheumatology/keaf536 (PMC12862366; doi:10.1093/rheumatology/keaf536)
Supplement: keaf536_Supplementary_Data [file keaf536_supplementary_data.docx]

**SUPPLEMENTARY APPENDIX**

**Table of contents**

| **Methods** | **2** |
| --- | --- |
| **Supplementary Figure S1:** Study design. A) core study; B) extension study | **3** |
| **Supplementary Figure S2: Recommended dosing regimen for administration of background SoC** | **4** |
| **Supplementary Table S1**: Eligibility criteria | **5** |
| **Statistical analysis** | **7** |
| **Supplementary Table S2**: Patient disposition | **9** |
| **Supplementary Table S3**: Baseline demographics and disease characteristics | **10** |
| **Supplementary Table S4**: Secondary endpoints (core study) | **12** |
| **Supplementary Table S5**: Proportion of patients achieving CRR in extension study | **12** |
| **Safety:** Serious adverse events (SAEs) suspected to be related to study treatment. | **12** |

**Methods**

**Study design**

**Core study:** This was a phase III, randomised, double-blind, placebo-controlled trial evaluating the efficacy and safety of secukinumab versus placebo in patients with active lupus nephritis (LN) receiving background standard of care (SoC) regimen at week 52. The SoC regimen consisted of induction therapy with mycophenolic acid (MPA; which refers to mycophenolate mofetil, or enteric-coated MPA sodium at equivalent doses [oral]), or cyclophosphamide (CYC) (i.v.), followed by maintenance therapy with MPA. The choice of background SoC induction therapy was at investigator's discretion. At randomisation, patients were stratified based on the SoC induction therapy they received during the study, MPA or CYC-based, to ensure a balanced representation in each of the treatment arms (secukinumab or placebo). In addition, corticosteroids were administered through i.v. pulses followed by tapering of oral daily doses (**Supplementary Figure S1A**). **Figure S2** depicts the recommended dosing regimen for administration of background SoC. **Extension study:** This was a planned three-year, phase 3, optional, open-label extension study to provide treatment with secukinumab for patients who completed core study and to obtain further data on long-term efficacy, safety and tolerability of secukinumab in patients with active LN. Investigators used their clinical judgement to decide if it might be beneficial, in terms of overall improvement and response to therapy, for patients to enter extension study. At week 104 of the core study, eligible patients who completed the assessments associated with the core study visit subsequently continued in the extension study on the dose of secukinumab 300 mg administered every four weeks. (**Supplementary Figure S1B**). The patients continued with the same dose of MPA as administered in the core study. The oral corticosteroid dosing regimen followed the same recommendations as outlined in the core study

**Supplementary Figure S1**: Study design:core study and extension study

**Alt text**: Figure showing the design of core and extenstions studies. The core study ran from screening to Week 104, comparing two treatment arms secukinumab + SoC and placebo + SoC. In the extension study, all eligible patients from both arms received secukinuamb upto Week 260, with follow-up continuing until Week 268.


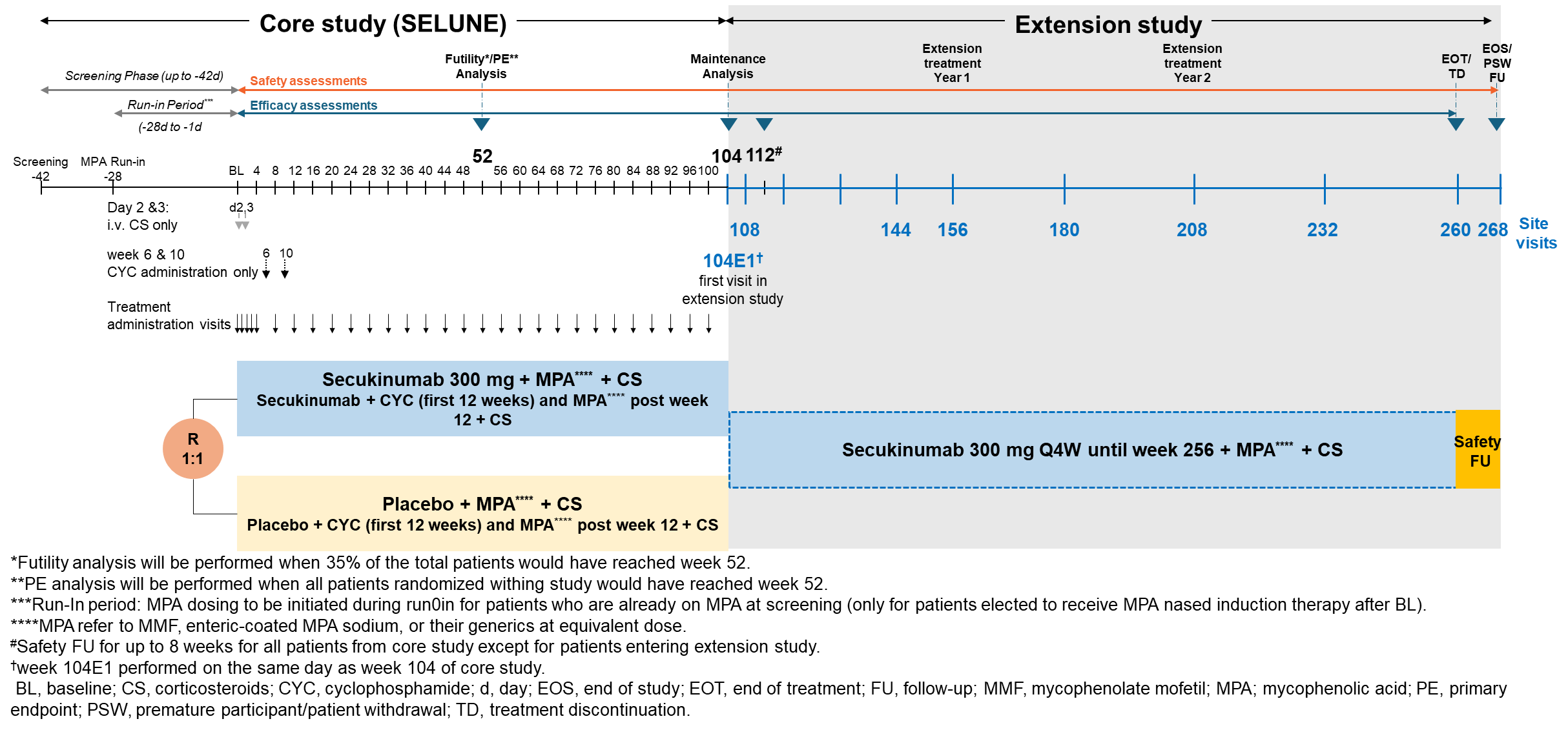


*Futility analysis was planned when 35% of the total patients would have reached week 52.

**PE analysis was planned when all patients randomised within study would have reached week 52.

***Run-In period: MPA dosing initiated during run-in for patients who were not already on MPA at screening (only for patients elected to receive MPA based induction therapy after BL).

****MPA refer to MMF, enteric-coated MPA sodium, or their generics at equivalent dose.

^#^Safety FU for up to 8 weeks for all patients from core study except for patients entering extension study.

^†^week 104E1 visit was performed on the same day as week 104 visit of core study.

BL, baseline; CS, corticosteroids; CYC, cyclophosphamide; d, day; EOS, end of study; EOT, end of treatment; FU, follow-up; MMF, mycophenolate mofetil; MPA; mycophenolic acid; PE, primary endpoint; PSW, premature participant/patient withdrawal; SoC, standard of care; TD, treatment discontinuation.

**Supplementary Figure S2:** Recommended dosing regimen for administration of background SoC.

**Alt text**: Flowchart depicting the dosing schedule of background SoC from run-in period through Week 24. Arrows depict the sequential flow through each stage.


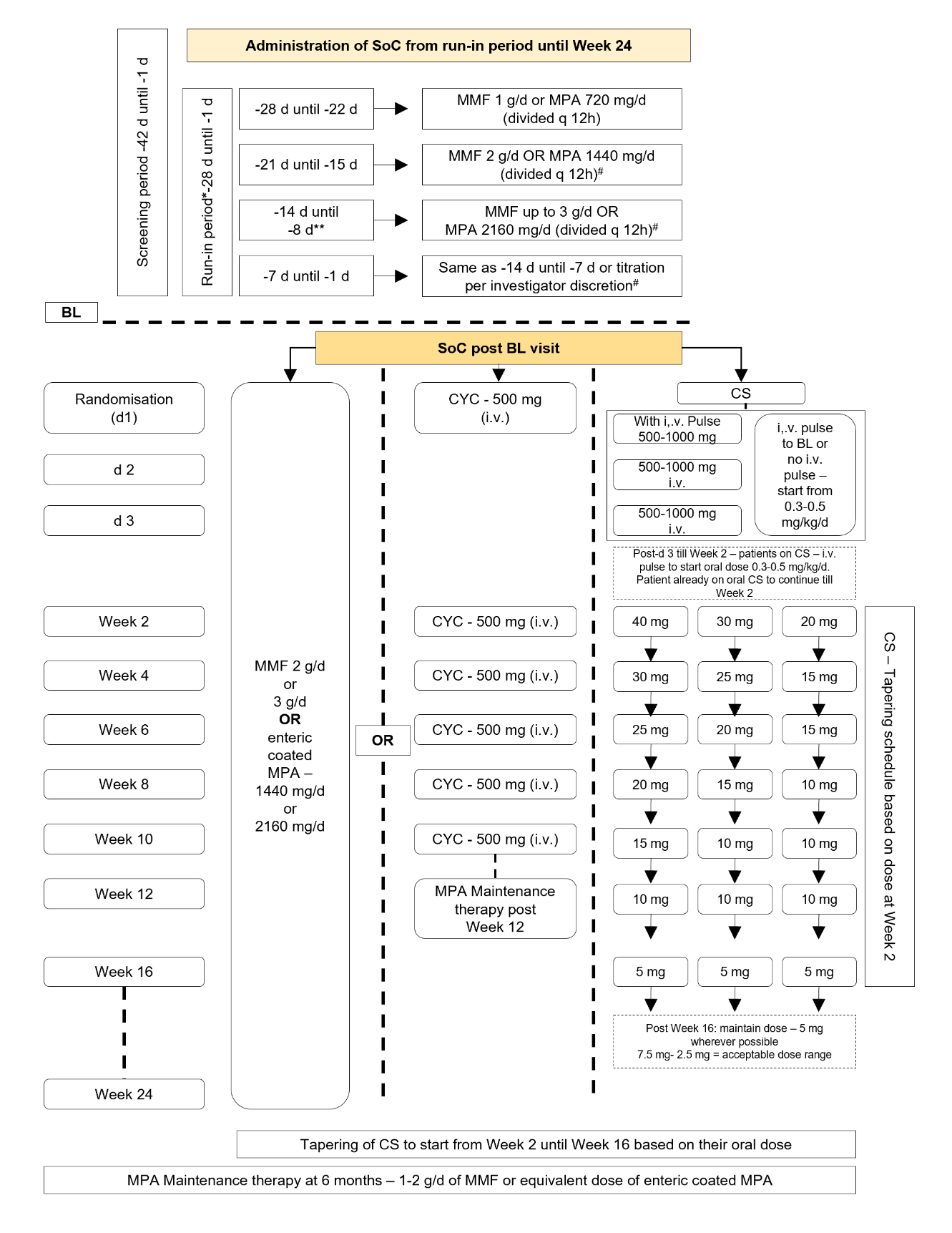


*Run-In period: MPA dosing initiated during run-in for patients who not were already on MPA at screening (only for patients elected to receive MPA based induction therapy after BL).

**Uptitration when requiered.

^#^If patient experienced adverse events, the dose could be titrated.

BL, baseline; CS, corticosteroids; CYC, cyclophosphamide; d, day; MMF, mycophenolate mofetil;
MPA; mycophenolic acid; SoC, standard of care.

**Supplementary Table S1: Eligibility criteria**

| **Core study** |
| --- |
| **Inclusion criteria**   1. Adult male and female patients aged 18-75 years old at the time of baseline 2. Confirmed diagnosis of:  - SLE with documented history of at least 4 of the 11 criteria for SLE as defined by the American College of Rheumatology   OR   - LN as the sole clinical criterion in the presence of antinuclear antibodies (ANA) or anti-dsDNA antibodies  1. Active LN, as defined by meeting the four following criteria:  - Biopsy within 6 months prior to screening visit indicating active glomerulonephritis of WHO or ISN/RPS Class III or IV LN [excluding III (C), IV-S (C) and IV-G (C)]; patients are permitted to have co-existing Class V. If no biopsy was performed within 6 months of screening, a biopsy was to be performed during the screening period - UPCR ≥1 mg/mg at screening - eGFR >30 mL/min/1.73 m^2^ by Chronic Kidney Disease Epidemiology Collaboration (CKD-EPI) - Active urinary sediment (presence of cellular casts [RBC or WBC casts]) or haematuria (> 5 RBC per high power field or above the laboratory reference range).  1. Patients must have currently been on MPA, or willing to initiate SoC induction therapy for LN according to the institutional practices using MPA or low-dose CYC in addition to corticosteroids 2. Patients must had been treated with anti-malarial agents (e.g., hydroxychloroquine), unless contra-indicated, and the dose had been stable for at least 10 days prior to randomisation 3. Able to provide signed informed consent |
| **Exclusion criteria**   1. Severe renal impairment as defined by i) Stage 4 CKD, or ii) presence of oliguria (defined as a documented urine volume <400 mL/24 h), or iii) ESKD that required dialysis or transplantation 2. Known intolerance/hypersensitivity to MPA, or oral corticosteroids, or any component of the study drug(s) 3. Patients received any other biologic immunomodulatory therapy within 6 months prior to screening, excluding belimumab where 3 months was acceptable 4. Previous exposure to secukinumab or any other biologic drug targeting IL-17 or the IL-17 receptor 5. Patients received any investigational drug within 1 month or five times the half-life of enrolment, whichever was longer 6. Receipt of more than 3000 mg i.v. pulse methylprednisolone (cumulative dose) within the 12 weeks prior to baseline 7. Treatment with a systemic calcineurin inhibitor (e.g., cyclosporine, tacrolimus) within 12 weeks prior to baseline 8. CYC use (i.v. or oral) within the month prior to baseline 9. Patients requiring dialysis within the previous 12 months before screening. 10. History of renal transplant 11. Any severe progressive or uncontrolled concurrent medical condition, including recent severe thromboembolic events, that, in the opinion of the principal investigator, renders the patient unsuitable for the trial 12. Active ongoing inflammatory diseases that might confound the evaluation of the benefit of secukinumab therapy, including inflammatory bowel disease 13. Presence of investigator-identified significant medical problems which at the investigator’s discretion would prevent the patient from participating in the study, including but not limited to the following: myocarditis, pericarditis, poorly controlled seizure disorder, acute confusional state, depression, and severe manifestations of neuropsychiatric SLE 14. Chest X-ray, computerised tomography (CT) scan, or MRI with evidence of ongoing infectious or malignant process, obtained within 3 months preceding the screening visit and evaluated by a qualified physician 15. History of chronic, recurrent systemic infections, active tuberculosis infection, or active systemic infections during the last two weeks (exception: common cold) prior to randomisation 16. Known infection with human immunodeficiency virus (HIV), hepatitis B or hepatitis C at screening or randomisation 17. History of lymphoproliferative disease or any known malignancy or history of malignancy of any organ system treated or untreated within the past 5 years, regardless of whether there was evidence of local recurrence or metastases (except for skin Bowen’s disease or basal cell carcinoma or actinic keratoses that had been treated with no evidence of recurrence in the past 12 weeks, carcinoma *in situ* of the cervix or non-invasive malignant colon polyps that had been removed) 18. Any of the following abnormal laboratory values on Screening evaluations as reported by Central Laboratory:  - Aspartate aminotransferase (AST), alanine aminotransferase (ALT), or  amylase >2.5 × ULN - Haemoglobin <8 g/dL - Neutrophils <1.0 × 10^9^/L - Platelet count <50 × 10^9^/L  1. Pregnant or lactating women or women of childbearing potential |
| **Extension study** |
| **Inclusion criteria**   1. Patient must have participated in core study and completed the entire treatment period up to week 104 of the core study 2. Patient must be deemed by the investigator to benefit from secukinumab treatment 3. Able to provide signed informed consent |
| **Exclusion criteria**   1. Any patient taking other concomitant immunomodulating agent(s) except secukinumab 2. Pregnant or lactating women or women of childbearing potential |

**Statistical analysis:**

**Sample size calculation:** The total planned sample size was 400 patients for the core study. Based on literature [1], the control response rate was assumed to be approximately 25%. Assuming a 15% treatment difference with a 40% response rate for secukinumab, the power for rejecting the null hypothesis for the primary endpoint (complete renal response) is 87%. Sample size for extension study was based on patients continuing from the core study.

**Interim analysis**: Two interim analyses were planned for the core study. However, since the futility analysis from the first interim analysis showed futile outcomes, the study was terminated early, and the second interim analysis was not conducted.

Summary statistics for continuous variables included N, mean, standard deviation (SD), minimum, lower quartile, median, upper quartile and maximum.

**Complete renal response (CRR):** A composite endpoint defined as

- - Estimated glomerular filtration rate (eGFR) ≥60 mL/min/1.73 m^2^ or ≥85% of baseline
  - 24-hour urine protein-to-creatinine ratio (UPCR) ≤0.5 mg/mg
  - Additionally, for the core study, patient must not have had treatment discontinuation before week 52 and must not have received >10 mg/day prednisone or equivalent for ≥3 consecutive days or for ≥7 days in total from week 44 through 52

**Partial renal response (PRR):** A composite endpoint defined as

- - eGFR ≥60 mL/min/1.73 m^2^ or ≥85% of baseline
  - ≥50% reduction in 24-hour UPCR to sub-nephrotic levels (≤3 mg/mg) compared to baseline

**Functional Assessment of Chronic Illness Therapy – Fatigue (FACIT-Fatigue©):** A 13-item questionnaire that assesses self-reported fatigue and its impact upon daily activities and function over the past week. The level of fatigue was measured on a 5-point Likert scale (0 = not at all, 1 = a little bit, 2 = somewhat, 3 = quite a bit, 4 = very much) [2,3].

**Short Form Health Survey (SF-36):** A survey evaluating individual subjects' health status, which also monitors and compares subjects' disease burden [4].

**Lupus Quality of Life (LupusQoL):** A disease-specific, 34-item, self-report questionnaire designed to measure the health-related quality of life (HRQoL) of subjects with SLE within 8 domains (i.e., physical health (8 items), emotional health (6 items), body image (5 items), pain (3 items), planning (3 items), fatigue (4 items), intimate relationships (2 items), and burden to others (3 items). Responses are based on a 5-point Likert scale where 0 (all of the time) to 4 (never) [5].

**Treatment-emergent adverse event**s (TEAEs): Events started after the first dose of study treatment or events present prior to the first dose of study treatment but increased in severity based on preferred term and on or before last dose date + 84 days).

**References**

1. Rovin BH, Teng YO, Ginzler EM, et al. Efficacy and safety of voclosporin versus placebo for lupus nephritis (AURORA 1): a double-blind, randomised, multicentre, placebo-controlled, phase 3 trial. *Lancet*. 2021;397:2070-80.
2. Cella DF, Tulsky DS, Gray G, et al (1993) The Functional Assessment of Cancer Therapy scale: development and validation of the general measure. J Clin Oncol. 1993; 11(3):570-9.
3. Yellen SB, Cella DF, Webster K, et al. Measuring fatigue and other anemia-related symptoms with the Functional Assessment of Cancer Therapy (FACT) measurement system. J Pain Symptom Manage. 1997;13:63-74.
4. Holloway L, Humphrey L, Heron L, et al. Patient-reported outcome measures for systemic lupus erythematosus clinical trials: a review of content validity, face validity and psychometric performance. Health Qual Life Outcomes. 2014;12:116.
5. Yazdany J. Health-related quality of life measurement in adult systemic lupus erythematosus: Lupus Quality of Life (LupusQoL), Systemic Lupus Erythematosus-Specific Quality of Life Questionnaire (SLEQOL), and Systemic Lupus Erythematosus Quality of Life Questionnaire (L-QoL). *Arthritis Care Res (Hoboken)*; 63:S413-9.

**Supplementary Table S2: Patient disposition**

| **Core study** | | | |
| --- | --- | --- | --- |
| **Disposition up to week 52*** | **SEC**  **N=91, n (%)** | **Placebo**  **N=91, n (%)** | **Total**  **N=182, n (%)** |
| **Completed study treatment** | 65 (71.4) | 68 (74.7) | 133 (73.1) |
| **^#^Discontinued study**  **treatment before week 52** | 25 (27.5) | 22 (24.2) | 47 (25.8) |
| **Primary reason for discontinuing study treatment before week 52** | | | |
| Adverse event | 7 (7.7) | 7 (7.7) | 14 (7.7) |
| Lack of efficacy | 6 (6.6) | 8 (8.8) | 14 (7.7) |
| Patient decision | 4 (4.4) | 2 (2.2) | 6 (3.3) |
| Study terminated by sponsor^†^ | 3 (3.3) | 2 (2.2) | 5 (2.7) |
| Death | 1 (1.1) | 1 (1.1) | 2 (1.1) |
| Protocol deviation | 1 (1.1) | 1 (1.1) | 2 (1.1) |
| Physician decision | 1 (1.1) | 1 (1.1) | 2 (1.1) |
| Pregnancy | 1 (1.1) | 0 (0.0) | 1 (0.5) |
| New therapy for study  indication | 1 (1.1) | 0 (0.0) | 1 (0.5) |
| **Extension study** | | | |
| **Disposition** | **SEC**  **N=16, n (%)** | **Placebo-SEC**  **N=15, n (%)** | **Total**  **N=31, n (%)** |
| **Discontinued study treatment before study termination** | 0 | 1 (6.7) | 1 (3.2) |
| **Primary reason for discontinuing study treatment** | | | |
| Study terminated by sponsor | 16 (100) | 14 (93.3) | 30 (96.8) |
| Adverse event | 0 | 1 (6.7) | 1 (3.2) |
| **Discontinued study** | 16 (100) | 15 (100) | 31 (100) |
| *Patients who had a chance to reach week 52 at time of study termination (SEC:91/137; Placebo: 91/138).  ^#^2 patients, one from SEC and one from placebo group, did not have a week 52 visit but preformed visits with study treatment administration after their planned week 52 visits.  ^†^Due to futile results of the futility analysis.  N, total number of patients; n, number of patients; SEC, secukinumab. | | | |

**Supplementary Table S3: Baseline demographics and disease characteristics**

| **Characteristic** | **Core study** | | | **Extension study*** | | |
| --- | --- | --- | --- | --- | --- | --- |
|  | **SEC**  **N=137** | **Placebo**  **N=138** | **Total**  **N=275** | **SEC**  **N=16** | **Placebo-SEC N=15** | **Total**  **N=31** |
| **Age, mean (SD)** | 34.1 (10.8) | 33.2 (11.3) | 33.6 (11.1) | 35.6 (8.3) | 30.6 (9.8) | 33.2 (9.3) |
| **Age group, n (%)** | | | | | | |
| <30 years | 55 (40.1) | 64 (46.4) | 119 (43.3) | 4 (25.0) | 7 (46.7) | 11 (35.5) |
| ≥30 years | 82 (59.9) | 74 (53.6) | 156 (56.7) | 12 (75.0) | 8 (53.3) | 20 (64.5) |
| **Gender, Female, n (%)** | 116 (84.7) | 124 (89.9) | 240 (87.3) | 14 (87.5) | 14 (93.3) | 28 (90.3) |
| **Race, n (%)** | | | | | | |
| White | 42 (30.7) | 52 (37.7) | 94 (34.2) | 2 (12.5) | 6 (40.0) | 8 (25.8) |
| Black or African American | 6 (4.4) | 9 (6.5) | 15 (5.5) | 0 | 1 (6.7) | 1 (3.2) |
| Asian | 67 (48.9) | 56 (40.6) | 123 (44.7) | 8 (50.0) | 8 (53.3) | 16 (51.6) |
| American Indian or Alaska Native | 21 (15.3) | 21 (15.2) | 42 (15.3) | 6 (37.5) | 0 | 6 (19.4) |
| **Ethnicity, n (%)** | | | | | | |
| Hispanic or Latino | 43 (31.4) | 51 (37.0) | 94 (34.2) | 6 (37.5) | 2 (13.3) | 8 (25.8) |
| Not Hispanic or Latino | 92 (67.2) | 86 (62.3) | 178 (64.7) | 10 (62.5) | 13 (86.7) | 23 (74.2) |
| **Time since first diagnosis of LN (years), mean (SD)** | 2.7 (4.2) | 3.5 (5.1) | 3.1 (4.7) | 3.7 (4.6) | 6.1 (7.1) | 5.0 (6.0) |
| **Patient's global assessment of disease activity (mm), mean (SD)** | 45.5 (27.2) | 43.8 (25.3) | 44.7 (26.2) | 31.3 (25.6) | 47.1 (25.6) | 39.0 (26.4) |
| **24-hour UPCR (mg/mg), mean (SD)** | 3.8 (3.4) | 4.1 (4.8) | 3.9 (4.1) | 2.8 (2.1) | 2.6 (2.4) | 2.7 (2.2) |
| **UPCR First Morning Void (mg/mg), mean (SD)** | 3.1 (2.7) | 2.9 (2.5) | 3.0 (2.6) | 2.2 (1.5) | 2.0 (1.9) | 2.1 (1.7) |
| **eGFR (mL/min/1.73 m^2^), mean (SD)** | 97.0 (32.1) | 96.7 (30.0) | 96.9 (31.0) | 93.1 (30.4) | 95.8 (22.4) | 94.4 (26.4) |
| **Serum creatinine (μmol/L), mean (SD)** | 77.2 (30.9) | 77.2 (30.1) | 77.2 (30.5) | 81.2 (35.6) | 75.3 (17.4) | 78.3 (28.0) |
| **Renal biopsy LN classification** | | | | | | |
| Class III focal LN (<50% glomeruli) with Class V membranous LN | 18 (13.1) | 20 (14.5) | 38 (13.8) | 1 (6.3) | 2 (13.3) | 3 (9.7) |
| Class III focal LN (<50% glomeruli) without Class V membranous LN | 17 (12.4) | 21 (15.2) | 38 (13.8) | 1 (6.3) | 1 (6.7) | 2 (6.5) |
| Class IV diffuse LN (≥50% glomeruli) with Class V membranous LN | 33 (24.1) | 32 (23.2) | 65 (23.6) | 2 (12.5) | 7 (46.7) | 9 (29.0) |
| Class IV diffuse LN (≥50% glomeruli) without Class V membranous LN | 68 (49.6)**^#^** | 65 (47.1) | 133 (48.4) | 12 (75.0) | 5 (33.3) | 17 (54.8) |
| *Baseline refers to the core study baseline.  ^#^One patient was diagnosed with LN class IV, but the date of the renal biopsy was missing  eGFR, estimated glomerular filtration rate; LN, lupus nephritis; N, total number of patients; n, number of patients with the characteristic; SEC, secukinumab; UPCR, urine protein-to-creatinine ratio. | | | | | | |

**Supplementary Table S4: Secondary endpoints (core study)**

| **Endpoint (observed data)** | **SEC**  **(N=137)** | **Placebo  (N=138)** |
| --- | --- | --- |
| **Proportion of patients achieving PRR at week 52, n (%)** | 41 (56.2) | 46 (63.9) |
| **Average daily dose of oral corticosteroids administered between weeks 16 and 52, mean (SD)** | 8.12 (6.38) | 7.48 (5.62) |
| **CFB in 24-hour UPCR at week 52, mean (SD)** | −2.20 (3.51) | −2.74 (5.94) |
| **CFB in FACIT-F at week 52, mean (SD)** | −2.0 (10.18) | −2.0 (9.51) |
| **CFB in SF-36 PCS at week 52, mean (SD)** | 3.41 (7.71) | 2.71 (6.89) |
| **CFB in LupusQoL physical health score at week 52, mean (SD)** | 7.62 (23.28) | 8.55 (20.59) |
| **Estimated probability of achieving CRR by week 52, (%)** | 43.6 | 45.2 |
| **Estimated probability of achieving PRR by week 52, (%)** | 64.9 | 59.0 |
| **Estimated probability of achieving a first morning void UPCR ≤0.5 mg/mg, (%)** | 65.9 | 61.1 |
| CFB, change from baseline; CRR, complete renal response; FACIT-F, Functional Assessment of Chronic Illness Therapy – Fatigue; PCS, physical component summary; N, total number of patients, n, number of patients with assessment in the specified visit; PRR, partial renal response; QoL, quality of life;  SF-36, medical outcome short form (36) Health Survey; SEC, secukinumab; UPCR, urine protein-to-creatinine ratio | | |

**Supplementary table S5: Proportion of patients achieving CRR in extension study**

| **Extension study** | | | |
| --- | --- | --- | --- |
| **Analysis visit** | **CRR**  **(using observed data)** | **SEC**  **N=16** | **Placebo-SEC**  **N=15** |
| **week 104E1** | n | 9 | 9 |
|  | Responder | 4 (44.4) | 5 (55.6) |
|  | 95% CI | 13.7, 78.8 | 21.2, 86.3 |
| **week 132** | n | 6 | 5 |
|  | Responder | 3 (50.0) | 5 (100) |
|  | 95% CI | 11.8, 88.2 | 47.8, 100 |
| CI, confidence interval; CRR, complete renal response; E1, first visit in extension study. N, total number of patients, n, number of patients with assessment in the specified visit; NRI, non-responder imputation; SEC, secukinumab. | | | |

**Safety: Serious adverse events (SAEs) suspected to be related to study treatment** During the core study, SAEs suspected to be related to study drug were comparable across both treatment groups. There were 8 SAEs reported in 8 patients (COVID-19, influenza, pneumonia, pyelonephritis acute, post herpetic neuralgia, diarrhoea infectious, pancytopenia and meningitis) in the secukinumab 300 mg group compared to 7 SAEs reported in 8 patients in the placebo group (pneumonia, herpes zoster, pyelonephritis acute, azotaemia, genital tract inflammation, chronic sinusitis and herpes zoster disseminated). All patients with SAEs related to study treatment recovered except the patient with pneumonia in the secukinumab 300 mg group.
